# The effects of a quality improvement project to reduce caesarean sections in selected private hospitals in Brazil

**DOI:** 10.1186/s12978-024-01851-9

**Published:** 2024-09-04

**Authors:** Maria do Carmo Leal, Rosa Maria Soares Madeira Domingues, Thaís Cristina Oliveira Fonseca, Tatiana Henriques Leite, Ana Claudia Figueiró, Ana Paula Esteves Pereira, Mariza Miranda Theme-Filha, Bárbara Vasques da Silva Ayres, Oliver Scott, Rita de Cássia Sanchez, Paulo Borem, Maria Carolina de Maio Osti, Marcos Wengrover Rosa, Amanda S. Andrade, Fernando Maia Peixoto Filho, Marcos Nakamura-Pereira, Jacqueline Alves Torres

**Affiliations:** 1 Department of Epidemiology and Quantitative Methods On Health, National School of Public Health, Oswaldo Cruz Foundation, Rio de Janeiro, Brazil; 2 National Institute of Infectious Disease Evandro Chagas, Oswaldo Cruz Foundation, Rio de Janeiro, Brazil; 3 Department of Statistical Methods, Federal University of Rio de Janeiro, Rio de Janeiro, Brazil; 4 Social Medicine Institute, State University of Rio de Janeiro, Rio de Janeiro, Brazil; 5 Oswaldo Cruz Foundation, Rio de Janeiro, Brazil; 6 Pasteur Hospital, Rio de Janeiro, Brazil; 7 Hospital Moinhos De Vento, Porto Alegre, Brazil; 8 Hospital da Luz, São Paulo, Brazil; 9 Institute for Healthcare Improvement, Boston, USA; 10 Hospital Israelita Albert Einstein, São Paulo, Brazil; 11 Institute Fernandes Figueira, Department of Obstetrics, Oswaldo Cruz Foundation, Rio de Janeiro, Brazil; 12 Cambridge Imperial, Cambridge, UK; 13 National Institute of Health for Women, Children and Adolescents Fernandes Figueira, Oswaldo Cruz Foundation (IFF/Fiocruz), Rio de Janeiro, Brazil; 14 Institute for Healthcare Improvement, Brasília, Brazil

**Keywords:** Bayesian analysis, Implementation analysis, Vaginal birth

## Abstract

**Background:**

Brazil is one of the countries with the highest rates of caesarean sections (CS), reaching almost 90% of births in the private sector. A quality improvement project called “Adequate Childbirth Project (PPA)” was conceived to reduce CS in the private sector. This project consisted of four primary components: “Governance”, “Participation of Women”, “Reorganization of Care” and “Monitoring”. This paper aims to evaluate: (1) which specific activities of the PPA had the largest effect on the probability of a woman having a vaginal delivery; (2) which primary component of the PPA had the largest effect on the probability of vaginal delivery and (3) which scenarios combining the implementation of different activities planned in the PPA had a higher effect on the probability of vaginal delivery.

**Methods:**

A sample of 12 private hospitals participating in the PPA was evaluated. We used a Bayesian Network (BN) to capture both non-linearities and complex cause-effect relations. The BN integrated knowledge from experts and data from women to estimate 26 model parameters. The PPA was evaluated in 2473 women belonging to groups 1–4 of the Robson classification, who were divided into two groups: those participating or not participating in the PPA.

**Results:**

The probability of a woman having a vaginal delivery was 37.7% higher in women participating in the PPA. The most important component of the project that led to an increase in the probability of vaginal delivery was “Reorganization of Care”, leading to a 73% probability of vaginal delivery among women in labor. The activity that had the greatest effect on the type of delivery was access to best practices during labor, with a 72% probability of vaginal delivery. Considering the 12 scenarios combining the different activities of the PPA, the best scenarios included: a non-scheduled delivery, access to information about best practices, access to at least 4 best practices during labor and respect of the birth plan, with an 80% probability of vaginal delivery in the best combinations.

**Conclusion:**

PPA has been shown to be an effective quality improvement program, increasing the likelihood of vaginal delivery in private Brazilian hospitals.

## Abstract (plain language summary)

**Introduction:** Brazil boasts one of the highest rates of caesarean sections (CS) globally, with nearly 90% of births in private facilities being delivered via CS. In response, the 'Adequate Childbirth Project – PPA' was launched as a quality improvement initiative aimed at curbing CS rates in private healthcare. Its goal is to improve the quality of childbirth and reduce the number of CS in private healthcare. The project has four main parts: 'Governance', 'Participation of Women', 'Reorganization of Care', and 'Monitoring'. **Method:** an evaluative study was conducted across 12 private hospitals involved in the PPA, involving 2473 women who were categorized into PPA participants and non-participants. They used a method called a cause-effect network to see which parts of the PPA helped more women have vaginal deliveries. **Results:** They found that women in the PPA were 37.7% more likely to have a vaginal delivery. Giving women access to good practices during labor and birth was really important. Also, 'Reorganization of Care' was the most important part of the project. It led to a 73% chance of vaginal delivery for women in labor. **Conclusion:** The PPA is effective in helping more women in private hospitals have vaginal deliveries. This means it's a good program for improving childbirth in Brazil's private hospitals.

## Background

The increase in caesarean section (CS) rates in the world is not a purely medical problem, as it involves social, cultural, and financial determinants. Therefore, strategies to reduce CS rates are complex and multifaceted [1, 2].

Brazil has one of the highest CS rates in the world and, since 2009, CS has become the main type of birth in the country. In the private sector, this issue is even more evident as this surgical procedure represents almost all births (88%) [3]. The excess CS in Brazil can be identified through the analysis of Robson groups, with most women falling into groups 1–4 [3], suggesting the absence of a clinical indication for CS. These avoidable procedures can largely be explained by cultural factors. For example, part of the pregnant population identifies CS as the safest way to give birth, allowing for control of the "surprises" that may arise with a vaginal delivery. Adding to this, many believe that delivery needs to be an event organized in advance and accurately [4].

Unnecessary CS is associated with adverse pregnancy outcomes. In Brazil, women without medical conditions had an almost three-fold higher risk of postpartum maternal death following CS compared to those with vaginal deliveries, mainly due to postpartum hemorrhage and complications of anesthesia [5]. Cases of maternal near miss were also 2.5 times more frequent in elective CS, even after adjusting for maternal complications, social conditions, and access to antenatal care [6]. Furthermore, Brazilian studies have shown a high early-term newborns (births at 37 and 38 weeks gestation), reaching 11.5% of the total number of births in the country. Of these, 39.3% of births are associated with pre-labor cesarean deliveries [7]. Early term births are associated with higher CS rates [8] and have an increased risk of adverse infant outcomes, especially among provider-initiated births [9].

There are two types of private hospitals in Brazil: those owned by health plan operators, and those not owned by health plan operators. In the latter type of hospital, the model of obstetric care constitutes an exclusive relationship between the doctor and the pregnant woman, which starts from antenatal care and continues until the time of delivery. In hospitals belonging to health plan operators, birth care may be provided by the same doctor who provided antenatal care or by the team on duty. In both types of hospitals, obstetric care is based around the obstetrician, with little participation from nurse-midwives [10].

Women's social movements in favor of vaginal deliveries and the change in the private sector obstetric care model resulted in discussions with the National Supplementary Health Agency (ANS), the regulatory agency of private health plans in Brazil- demanding action to address the CS problem [11, 12]. In 2014, women demanded effective action to reduce unnecessary CS in private hospitals in Brazil, through a public civil lawsuit filed by the Federal Public Ministry against the ANS. In response to these social and legal demands, the ANS devised a program to improve the quality of births, beginning with the most respected hospitals [13]. This project called the “Adequate Childbirth Program (PPA)”, was conceived in partnership with the well-respected Hospital Israelita Albert Einstein—(HIAE), the Institute for Healthcare Improvement (IHI), and the Ministry of Health.

The main objective of the PPA was to identify innovative and viable models of care for labor and childbirth, which value vaginal delivery and reduce the frequency of excessive CS in the supplementary health system [13]. To achieve this objective, the PPA targeted improvements across four components: (1) “Governance”: forming a coalition between the leadership in the health sector, aligning quality and safety in labor and childbirth care; (2) “Participation of Women”: empowering women and families so they actively participate in the entire process of pregnancy, delivery, and postpartum care; (3) “Reorganization of Care”: reorganizing the model of childbirth care to favor the physiological evolution of labor and ensuring that CS is based on clinical criteria; (4) “Monitoring”: structuring information systems to allow for lifelong learning [13]. For each component, the PPA team defined a range of activities, to be tested at a smaller scale, before being adapted to the hospital context and implemented.

The PPA was enacted in three phases. Phase 1, which was developed between 2015 and 2016, aimed to test the proposed interventions in 35 participating public and private hospitals, involving 19 health plan operators. Phase 2 was characterized by extending the project to a wider variety of providers and health operators. Finally, phase 3, which was launched in October 2019, aims to disseminate effective strategies on a large scale, with the possibility of including the entire set of maternity hospitals and operators in Brazil [14].

The PPA model of care is based on scientific evidence [15] and on 2 successful strategies for reducing caesarean sections in Brazilian private hospitals [11, 16]. However, there was a knowlegde gap about the activities that would have the greatest effect on cesarean section rates, which would be prioritezed by health managers, health professionals and health policy makers. In real life, the interaction of multiple components within a complex system such as healthcare makes it difficult to identify the importance of every single component with currently existing methods. [17]. In 2017, researchers from the Oswaldo Cruz Foundation conducted an external evaluative study called “Healthy Birth” to evaluate the implementation and effects of the PPA. Using data from the “Healthy Birth” study, this paper has three main objectives: (1) to evaluate which specific activities of the PPA have the largest effect on the probability of women giving birth via vaginal delivery; (2) to evaluate which component of the PPA has the largest effect on the probability of vaginal delivery, and (3) to evaluate different scenarios of the PPA implementation on the probability of vaginal delivery.

## Methods

### Study design

The “Healthy Birth” is a hospital-based evaluative study using a mixed-methods approach, with a cross-sectional design in the quantitative component. Quantitative data were collected in two stages: the first from March 2017 to August 2017, 6 to 8 months after the end of the first phase of PPA; and the second from May 2018 to August 2018. The first data collection period aimed at assessing the degree of implementation of PPA, while the second aimed at assessing the sustainability of the implementation of the PPA 1 year later. In this analysis, we will use data from the quantitative component collected during the first data collection period of the “Healthy Birth” study.

### Sample design and study population

We selected 12 of the 23 private hospitals included in the first phase of the PPA for this study. Neither the researchers nor those responsible for the obstetric units knew which hospitals would be selected before and during this phase of the PPA implementation. The sample was selected based on three criteria, each of which had the potential to influence the implementation of activities related to the project. Criteria included: hospital location (according to Brazilian regions, due to cultural differences and the organization of services); type of hospital (hospitals owned or not owned by health plan operators, due to economic interests in reducing cesarean section); and hospital performance (hospital performance classified as “good” or “poor”, according to the evaluation by the PPA coordination team, to evaluate the best and worst hospitals in the reduction of cesarean sections) [13]. Based on these criteria, we formed 8 possible strata, with a balanced distribution of the 12 hospitals. However, it was not possible to select hospitals from 2 strata, due to the absence of hospitals in the North/Northeast region that met the selection criteria [13].

Within each of the 12 hospitals selected for the study, the intended sample size was 400 women. This size was chosen to detect a 10% reduction in the proportion of CS, considering an estimated proportion of 50%, with 80% power and a 5% significance level. All women admitted to the selected maternity hospitals who had a live birth (of any gestational age or birth weight) or a stillbirth (with gestational age ≥ 22 weeks and/or birth weight ≥ 500 g), were eligible for the study. Exclusion criteria included women who gave birth prior to hospital admission and women with extreme communication difficulties (foreigners who could not understand Portuguese, deaf-mute women, and women with mental or neurological diseases suffering severe cognitive impairment).

In this analysis, only women from groups 1–4 of the Robson classification were included (primiparous or multiparous women with single, cephalic, term pregnancies without previous CS) [18]. This criterion was adopted to improve the comparability between groups, as there was a higher proportion of women with previous CS (group 5 of the Robson classification) in the group not exposed to the PPA. Therefore, 2393 women, representing 49.1% of the total intended sample, were included in the study. Additionally, at each hospital, the hospital director, or alternatively the head of Obstetrics or the head of nursing at the obstetric center, was also included in the study. This resulted in 12 management interviews.

### Data collection

The management interview occurred at the beginning of the fieldwork period in each hospital and focused on the structure and processes of the hospital, taking into account the four driving components of the PPA.

Women were interviewed during hospitalization for childbirth care to avoid recall and survival bias. They did not receive prior information about the evaluative research and were invited to participate during hospitalization, when they were presented with the research objectives and procedures. Face-to-face interviews with eligible women were carried out during the post-partum period (at least 6 h after vaginal delivery and 12 h after CS) by trained interviewers, mostly nurse-midwives. The interview included questions on maternal identity; socio-economic status; previous obstetric history; maternal anthropometric data; prenatal care; illnesses and medication during gestation, labor, and birth; and evaluation of childbirth care received by the woman and the newborn. We also extracted data from the medical records of the women and neonates after hospital discharge.

We used electronic data collection instruments that were developed for this study and are available at Torres et al. [13]. Women and health professionals signed the free and informed consent form before the interview.

### Theoretical model

**Fig. 1:**
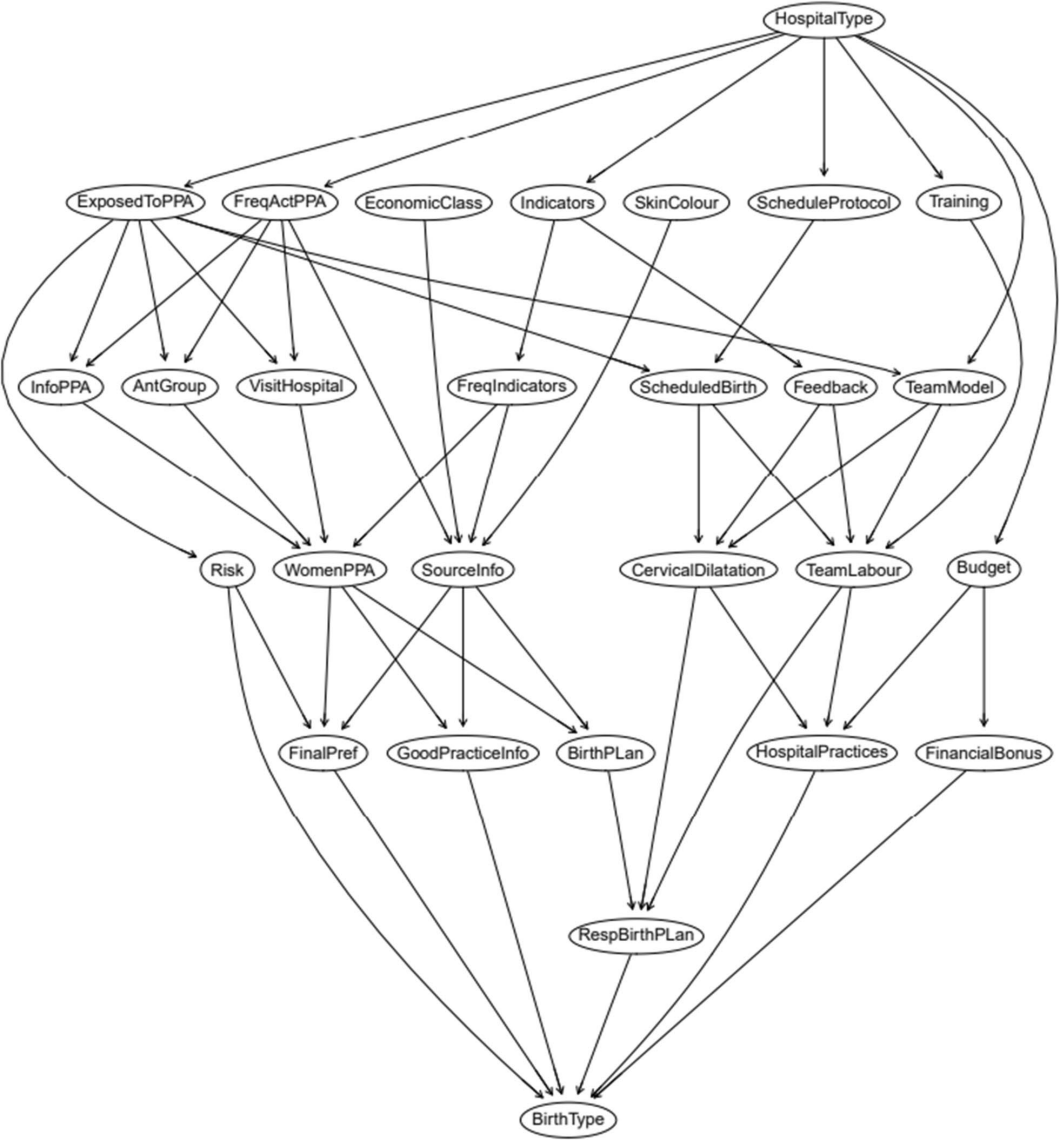
The Birth Network used for data analysis

To assess the effect of the PPA on CS, we used “The Birth Network” (Fig. 1)—a theoretical model developed by the research team after consulting experts on the topic, including obstetricians, nurse-midwives, and epidemiologists. The network considered the four components of the PPA (“Governance”, “Participation of Women”, “Reorganization of Care”, “Monitoring”) and potential confounders of the effects of the PPA in reducing CS rate. All the variables used in The Birth Network are described in Table 1. The “Governance” component included activities that would favor the implementation of the quality improvement project in the hospital, such as a specific budget for maternal and child care, financial incentives to reduce cesarean sections and training of the hospital team. The “Participation of women” component included actions such as educational activities and campaigns, disseminating information about the project, visits to participating hospitals, and the development of a birth plan, while the “reorganization of care” component included changes in the hospital environment, access to non-pharmacological methods for pain relief, equipment for births in vertical positions, inclusion of nurse-midwives in childbirth care and implementation of clinical guidelines. Finally, the “monitoring” component included the use of health indicators to monitor the planning and evaluation of activities. Note that the outcome of interest in the network is “Birth Type”, which is at the lowest level of the model, thus being affected by all the variables in the network.

**Table 1:** Birth network variables

Variable name	Variable description	Answer categories	Source of data
*I—Governance*			
Training (G1)	Whether the hospital staff participated in training offered by the Open School Institute for Healthcare Improvement, Sofia Feldman Hospital, and Albert Einstein Hospital	“All”—for three instances of training or “Two or less”—for 0 to 2 instances of training	Management interview
Financial bonus (G2)	Whether the hospital uses a financial bonus strategy to implement protocols and routines	“Yes” or “No”	Management interview
Budget (G3)	Whether the hospital has a budget to improve maternal and childcare	“Yes” or “No”	Management interview
*II) Participation of women*			
Good practice info (W1)	Whether women received information during pregnancy about: 1) signs of labor, 2) signs of risk, and 3) best practices during labor	“None”—received no information or “Yes”—received at least one category of information	Interview with women
Final Pref (W2)	Women’s final preference of the type of delivery	“Vaginal”, “Caesarean” or no preference	Interview with women
Info PPA (W3)	Whether women knew that the hospital was participating in the PPA	“Yes” or “No”	Interview with women
Women PPA (W4)	Variable composed of three items: 1) if the participation of the hospital in the PPA was important for the woman’s choice of this hospital for delivery; 2) if the woman visited the hospital before delivery; 3) if the woman participated in a hospital antenatal group	“All”—to women who responded “yes” to the three questions or “Two or less”—to women who responded “yes” to two or fewer questions	Interview with women
Source Info (W5)	Whether information about best practices was provided by the hospital/insurance company, or from other sources	“Hospital/Health Insurance Company” or “Other sources”	Interview with women
Freq Act PPA (W6)	Frequency of publication of the PPA activities to women/clients	“Regular” or “Non-regular”	Management interview
Ant group (W7)	Whether women were offered the opportunity to participate in an antenatal group activity	“Yes” or “No”	Interview with women
Visit hospital (W8)	Whether women were offered the opportunity to visit the hospital prior to delivery	“Yes” or “No”	Interview with women
Birth plan (W9)	Whether a birth plan was prepared by the woman	“Yes” or “No”	Interview with women
*III) Reorganization of care*			
Team model (R1)	Type of healthcare team providing labor and childbirth care	“Hospital staff”, “External and Hospital staff” or “External staff”	Interview with women
Team labor (R2)	Type of healthcare worker providing labor and childbirth care	“Doctor”, “Nurse” and “Doctor and Nurse”, or “No labor”	Interview with women
Schedule protocol (R3)	Existence of a protocol for scheduling CS according to gestational age at birth	“Yes” or “No”	Management interview
Scheduled birth (R4)	Whether the birth was scheduled	“Yes” or “No”	Interview with women
Cervical dilatation (R5)	Cervical dilatation upon hospital admission	“No labor”, “ < 4 cm” or “ >= 4 cm”	Medical records
Resp birth plan (R6)	Whether the birth plan was respected	“Respected”, “Not respected” or “No birth plan”	Interview with women
Hospital practices (R7)	Whether access to best practices during labor (oral fluids, freedom of movement, shower, non-pharmacological methods of pain relief) was provided	“ < 4 items”, “ >= 4 items” or “No Labor”	Interview with women
*IV) Monitoring*			
Indicators (M1)	Whether the hospital monitors the following perinatal indicators: CS rate, CS rate by Robson group, childbirth care by nurse-midwives, vaginal birth with episiotomy, admission to Neonatal Intensive Care Unit and proportion of early-term births (37–38 gestational weeks)	<= 4 items, 5 or 6 items	Management interview
Feedback (M2)	Identifying which professionals gathered feedback on the results of the perinatal indicators	“Each doctor individually”, “Doctors and team” or “Doctors, team and user”	Management interview
Freq indicators (M3)	Frequency of feedback regarding perinatal indicators	“No frequency”, “Regular”, “Irregular”, “Hospital does not monitor indicators”	Management interview
*V) Confounders*			
Economic class	Brazilian economic classification [40]	A1/A2, B1/B2, or C1/C2 (where “A” represents the highest economic class)	Interview with women
Skin colour	Self-reported skin color	“White” or “Non-white”	Interview with women
Risk	Presence of at least one of the following conditions: hypertensive syndrome, diabetes, placenta previa, placental abruption, infection, oligodramnia, polydramnia, intrauterine growth restriction	“Yes” or “No”	Women’s medical records
*VI) Exposed*			
Exposed To PPA model	Whether the woman was targeted by the PPA model of care	“Yes” or “No”	Management Interview
*VII) Context*			
Hospital	Whether the hospital belonged to a health insurance company	“Yes” or “No”	Management interview
*VIII) Outcome*			
Birth type	Type of birth	“Vaginal/forceps” or “Caesarean section”	Interview with women

The analyses were carried out following the classification of women into two groups, the first being those participating in the model of care recommended by the PPA, called “Exposed to the PPA model”, with the second being the population exposed to the “Standard of care model” in private hospitals. The “Exposed to the PPA model” group was defined per participating hospital. In two of the participating hospitals, the target population of the PPA was composed of all primiparous women. In a further two hospitals, the population included women in Robson's groups 1–4. Finally, in the remaining eight hospitals, the population was comprised of women admitted by the hospital's on-duty staff. The “Exposed to PPA model” group would theoretically be exposed to the activities advocated by the quality improvement project, including access to information during pregnancy; visits to the maternity hospital; preparation of a birth plan by the pregnant woman; encouragement of labor; assistance of labor and childbirth care in a collaborative doctor-nurse model; and use of best practices [13].

Women in the “Standard of care model” were assisted according to the current practice in Brazilian private hospitals, which is characterized by having the same doctor responsible for prenatal care and childbirth care; low participation of nurse-midwives; a high proportion of antepartum CS; and intensive use of interventions in labor and childbirth care [19].

### Statistical analysis

The model used to analyze the data in this study is a Bayesian Network (BN), which is a directed acyclic graphical model that can represent causal interactions among variables in a multivariate problem. The divide and conquer strategy of a BN alleviates the curse of dimensionality for large systems such as the Birth Network. The model is characterized by a topology G (a graph structure defining the directions of the arcs) and the conditional distribution for Y_i_ | Y_C(i)_, p_ij_ (representing the strength of the causal links), with the variables being denoted by Y_i_, with i = 1,…, 26, levels for the parent nodes represented by z_j_ and the probabilities of interest by p_ij_. The proposed BN approach uses expert judgment to elicit the Birth Network structure and the data are used to estimate the probabilities of events within the network. The model is decomposed in conditional local distributions for each variable, and in our context, a multinomial model is assumed for each node in the network.

$$Y_i |Y_{c\left( i \right)} = Z_{j } , P_{ij } \ Mult\left( {M_{ij} , P_{ij} } \right)$$

The parameters are estimated via Bayesian inference with posterior Dirichlet distributions. Predictive probabilities of scenarios are computed via simulation. In particular, logic sampling was used as the simulation method. The basic idea for BN sampling is to traverse the network in topological order, visiting parents before children, and to generate a value for each visited node according to the conditional probability of that node. Furthermore, we compute expected probabilities for each scenario representing possible decisions. For more details about the method used for estimation, see Heckerman et al. [20] and Nagarajan et al. [21].

Based on this analysis, we initially described the predicted probability for all of the variables making up the network, according to the information from women in the two groups: “Exposed to the PPA model” and “Standard of Care model”. We then described the differences in the probability of vaginal delivery for the two groups for all variables in the network, investigating each variable individually for its impact on the outcome (vaginal delivery). To do this, the variable under test was fixed in each answer type in turn, while the other variables had their probabilities estimated from the observed data. For example, in order to predict how much the variable “Birth Plan” impacts the probability of vaginal delivery, we first considered that no women had a birth plan, and then calculated the probability of vaginal delivery in this scenario using observed data to estimate probabilities for each of the other variables. Subsequently, we considered that all women made a birth plan and estimated the probability of vaginal delivery in this alternative, more favorable scenario. Using this, we calculated the difference between the worst and best-case possibilities, considering only the variation of the test variable. The greater the difference, the greater the effect of that variable on the outcome. Equal estimation was done to assess which of the four key components previously mentioned had the greatest effect on the probability of a vaginal delivery. For this analysis, all indicators of each component were fixed in their best (high level) and worst (low level) categories so that the differences could be calculated. Finally, simulations were run to calculate the predicted probability of vaginal delivery in different scenarios.

We opted for fixed sample size for logistical reasons and used sample weights to deal with variations in number of deliveries per year among the selected hospitals in othes analysis. However, in Bayesian statistics we assume that conditional on the model parameters and the hospital type variable, the births are interchangeable. Therefore, under the Bayesian approach, it is not necessary to use weigthing and calibration procedures. All analysis was conducted in R.

## Results

In the “Participation of Women” component, women who participated in the PPA were more likely: to receive information about best practices in labor and birth during prenatal care; to participate in an antenatal group; to participate in activities related to the PPA; and finally, to have a birth plan (Table 2).

**Table 2:** Predicted probabilities of all variables in the birth network

Indicator	Total	Exposed to the PPA model		Standard care group		Significance level^1^
	n	n	% (CI 95%)	n	% (CI 95%)	
*Governance*						
Training (G1)		Management interview				
All	2					
Two or less	10					
Financial bonus (G2)		Management interview				
No	8					
Yes	4					
Budget (G3)		Management interview				
No	4					
Yes	8					
*Participation of women*						
Good practices info (W1)						
None	497	292	19.8 (19.1–20.5)	205	20.6 (19.3–21.7)	*
At least one	1976	1379	80.2 (79.4–80.9)	597	79.4 (78.1–80.6)	
Final pref (W2)						
Vaginal	1468	1171	60.1 (58.9–60.9)	297	59.3 (57.6–60.5)	*
Caesarean/no preference	1005	500	39.9 (39.1–40.7)	505	40.7 (39.5–41.9)	
Info PPA (W3)						
No	1498	1030	39.8 (39.8–39.4)	468	35.8 (34.5–37.0)	**
Yes	975	641	60.2 (59.3–61.1)	334	64.2 (62.6–65.5)	
Women PPA (W4)						
No	1310	845	51.9 (51.0–52.8)	465	58.3 (56.9–59.6)	**
Yes	1163	826	48.1 (47.0–49.1)	337	41.7 (40.6–42.9)	
Source info (W5)						
Hospital/insurance	184	132	11.2 (10.6–11.7)	52	10.6 (9.8–11.3)	*
Others	2289	1539	88.8 (88.2–89.6)	750	89.4 (88.5–90.1)	
Freq Act PPA (W6)		Management interview				
Regular	9					
Non regular	3					
Ant group (W7)						
No	1516	1016	60.0 (59.1–60.9)	500	68.3 (66.9–69.2)	**
Yes	957	655	40.0 (39.0–41.0)	302	31.7 (30.5–33.1)	
Visit hospital (W8)						
No	1097	733	56.3 (55.1–57.1)	364	45.9 (44.6–47.2)	**
Yes	1376	938	43.7 (42.8–44.7)	438	54.1 (52.9–55.4)	
Birth plan (W9)						
Birth plan	288	215	26.4 (25.5–27.3)	73	18.1 (16.9–19.1)	**
No birth plan	2185	1456	73.5 (72.7–74.4)	729	81.8 (80.8–82.6)	
*Reorganization of Care*						
Team model (R1)						
Hospital staff	972	940	56.2 (55.1–56.9)	32	3.9 (3.4–4.5)	**
External/hospital staff	266	160	9.6 (9.0–10.0)	106 664	13.4 (12.5–14.1)	
External	1235	571	34.1 (33.2–35.0)		82.7 (81.6–83.7)	
Team labor (R2)						
Doctor	579	476	23.7 (22.9–24.6)	103	12.0 (11.1–12.7)	**
Doctor/nurse	556	484	31.2 (30.3–32.0)	72	18.4 (17.3–19.3)	
No labor	1338	711	45.1 (44.3–46.0)	627	69.6 (68.3–70.7)	
Schedule protocol (R3)		Management interview				
No	1					
> 39 weeks	9					
> 40 or 41 weeks	2					
Scheduled birth (R4)						
No	1546	1263	74.8 (74.1–75.6)	519,283	64.4 (63.1–65.5)	**
Yes	927	408	25.2 (24.3–25.9)		35.6 (34.1–36.6)	
Cervical dilatation (R5)						
< 4	238	206	12.3 (10.9–13.5)	32	11.1 (9.8–13.9)	**
>= 4	897	754	45.2 (44.3–46.0)	143	19.3 (18.0–20.4)	
No labor	1338	711	42.5 (41.7–43.6)	627	69.6 (68.3–70.7)	
Respect birth plan (R6)						
Respected	251	191	16.5 (15.7–17.1)	60	11.5 (10.6–12.2)	**
Not respected/partially	37	24	10.0 (9.25–10.3)	13	6.7 (6.0–7.4)	
No birth plan	2185	1456	73.5 (72.7–74.2)	729	81.8 (80.8–83.1)	
Hospital practices (R7)						
< 4 recommended	478	380	28.4 (27.5–29.3)	98	15.6 (14.6–16.6)	**
>= 4 recommended	657	580	26.4 (25.0–27.6)	77	14.8 (13.3–16.3)	
No labor	1338	711	45.2 (44.3–46.0)	627	69.6 (68.3–70.7)	
*Monitoring*						
Indicators (M1)		Management interview				
<= 4	2					
> 4	10					
Feedback (M2)		Management interview				
Each doctor	6					
Doctors + Team	3					
Doctors + Team + User	3					
Freq Feedback (M3)		Management interview				
No frequency	6					
Regular	1					
Irregular	3					
Not monitor indicators	2					
*Confounders*						
Economic class						
A	467	375	18.9 (18.1–19.6)	92	19.2 (17.8–20.0)	NS
B	1422	904	57.5 (56.6–58.4)	218	57.3 (56.2–58.7)	
C/D	584	392	23.5 (22.8–24.4)	192	23.5 (22.3–24.6)	
Skin colour						
White	1541	1102	62.4 (61.4–63.3)	439	62.3 (60.9–63.7)	NS
Non-white	932	569	37.6 (36.7–38.8)	363	37.7 (36.3–39.3)	
Risk						
No	1887	1296	77.5 (76.8–78.3)	591	73.7 (72.5–74.8)	**
Yes	586	375	22.5 (21.6–23.2)	211	26.3 (24.9–27.3)	
*Outcome*						
Birth type						
Vaginal/forceps		699	37.7 (36.7–38.6)	121	24.5 (23.5–25.7)	**
Caesarean section		972	62.3 (60.8–63.8)	681	75.5 (74.2–76.8)	

^1^Significance level: NS = the Bayesian Confidence Interval coincide indicating the effects are equal; ** high significance = the Bayesian Confidence Interval do not intercept indicating the probability of equal effects is small or zero; *low significance = the Bayesian Confidence Interval intercept partially indicating the probability of equal effects is moderate

As for the "Reorganization of Care" component, women in the "Exposed to the PPA" model were more likely: to be cared for by the team on duty; to have a nurse-midwife present during labor and delivery; to be admitted when in active labor; to not have scheduled the delivery; to have their birth plan respected, and to have access to good practices that promote vaginal delivery (Table 2).

Indicators about “Governance” and “Monitoring” were assessed using information provided by the hospital manager about the organization as a whole, rather than on the level of individual women. Therefore, it was not possible to evaluate these indicators according to the groups “Exposed to the PPA Model” and “Standard of Care model” (Table 2).

Finally, women in the “Exposed to the PPA model” group had a 37.7% higher probability of a vaginal delivery when compared to women in the “Standard Care Group”. The two groups were similar in socioeconomic characteristics but diverged in CS risk (Table 2).

**Fig. 2:**
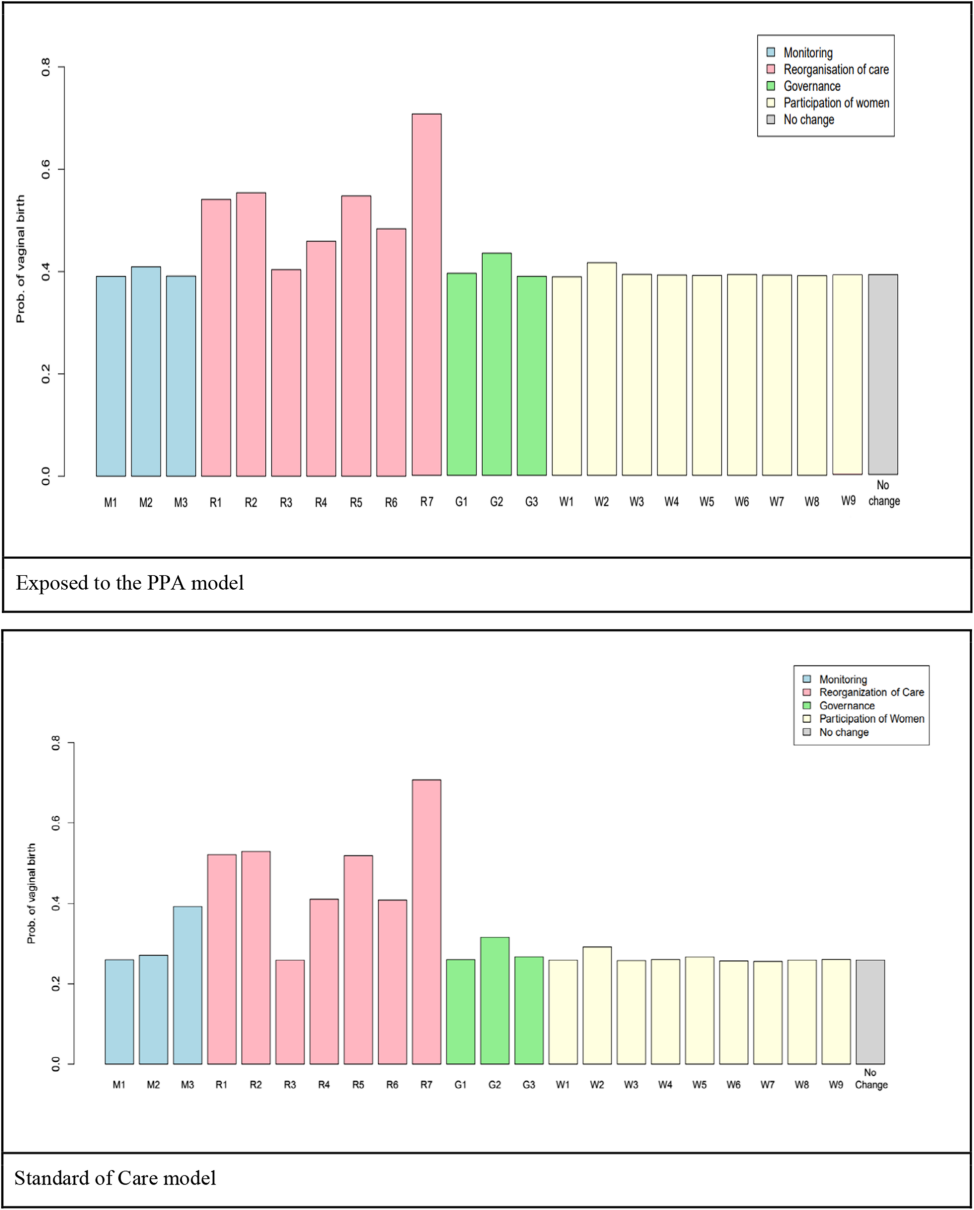
Individual effect of each variable in The Birth Network on the probability of vaginal delivery, for the women “Exposed to the PPA model” and “Standard of Care model”. *Note*: please see Table 1 for definition of variables

In Fig. 2, the individual effect of each variable in the Birth Network on the outcome of “vaginal delivery” is presented. In general, larger effects were observed in the group “Exposed to the PPA model”. In this group, the variable that presented the largest effect on the birth type was “Hospital Practices” (R7) with probability of vaginal delivery being 72%, while the variable that presented the lowest effect was “Antenatal Group” (W7), with a probability of vaginal delivery of 39%.

**Fig. 3:**
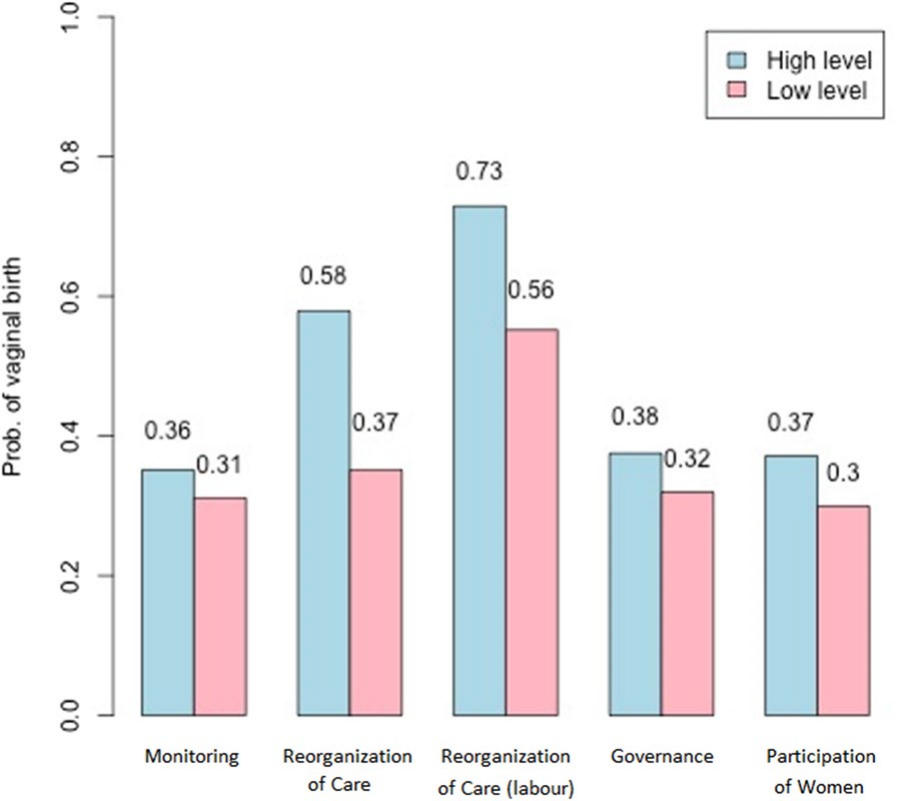
Effect of each driving component of the PPA on the outcome of “vaginal delivery”. *Note*: High level = all indicators of each component fixed in their best categories; Low level = all indicators of each component fixed in their worst categories

Figure 3 displays the effect of each driving component of the PPA on the outcome of “vaginal delivery” for all women in the study. The differences in the probability of vaginal delivery were 5% (“Monitoring”), 21% (“Reorganization of Care”), 17% (“Reorganization of Care” in women with labour), 6% (“Governance”), and 7% (“Participation of Women”), indicating that larger effects were obtained for the Reorganization of Care, especially for women with labor (probabilities of 73% and 44% for women with high and low levels, respectively).

**Fig. 4:**
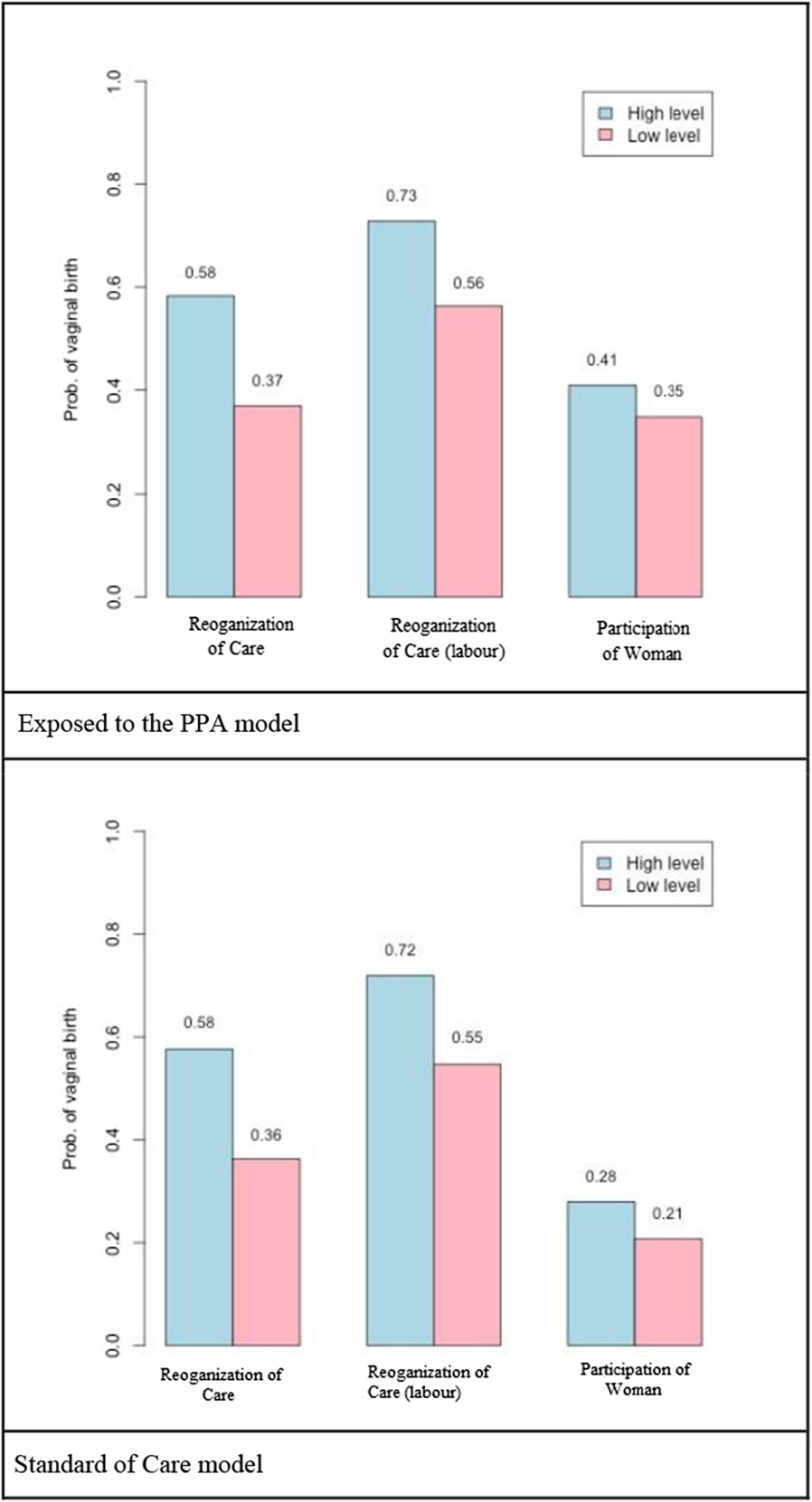
Effect of the components “Reorganization of care” and “Participation of Women” on the outcome of “vaginal delivery” among women in the “Exposed to PPA model” and in the “Standard of Care model”. *Note*: High level = all indicators of each component fixed in their best categories; Low level = all indicators of each component fixed in their worst categories

Figure 4 displays the effect of each PPA component on the outcome of “vaginal delivery” among women in the “Exposed to the PPA model and in the “Standard of care Model” groups, except for the PPA components “Governance” and “Monitoring”, which were assessed for the entire hospital.

In both groups of women, the probability of vaginal delivery was similar for the “Reorganization of Care” component, for the analysis including all women and for the analysis including only women who underwent labor. A larger difference was predicted for the component “Participation of Women” both in the scenarios of high or low level of implementation, with the probabilities of vaginal delivery at 41% and 28% for the “Exposed to the PPA model” and “Standard of care model” respectively, in the high level of implementation scenario.

**Fig. 5:**
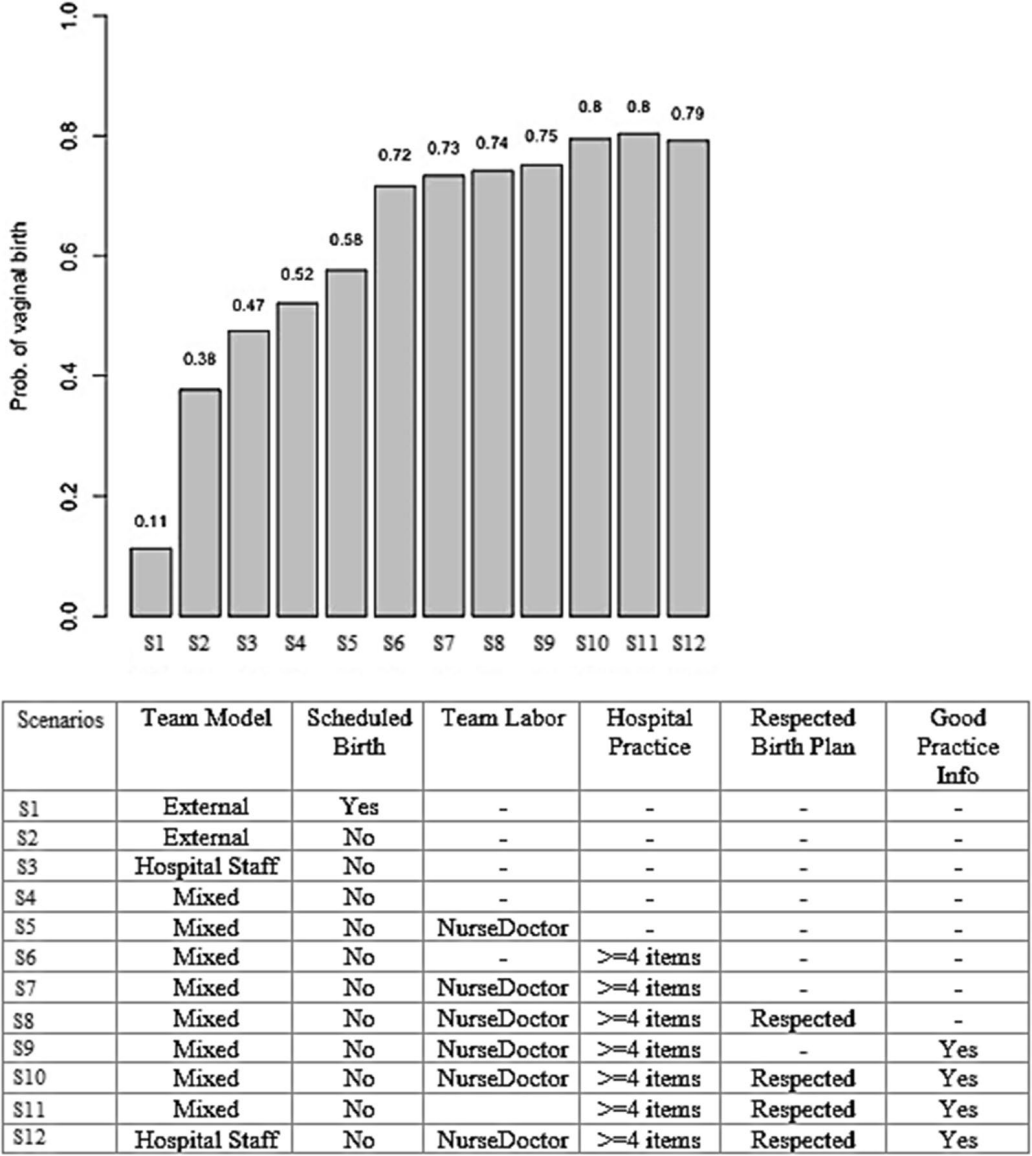
Probability of vaginal delivery according to different scenarios. *Note*: please see Table 1 for definition of variables

Figure 5 presents the probability of vaginal delivery according to 12 scenarios, each including a different combination of PPA recommended activities. The worst scenario (scenario 1) used an external team and a scheduled birth, with a probability of vaginal delivery of 11%. The best scenarios were S10, S11, and S12, which achieved probabilities of vaginal delivery of 80%, 80%, and 79%, respectively. These three scenarios all include: not having a scheduled delivery, access to information on best practices, access to at least 4 best practices during labor, and a respected birth plan differing on the team model (mixed or staff team) and on the presence of a nurse-midwife.

## Discussion

This study shows that there was a 37.7% increase in the probability of vaginal delivery in women in the “Exposed to the PPA model” group. It is noteworthy that the hospitals that participated in this quality improvement project had high CS rates—between 80 and 90% of their total deliveries. Given these higher rates, an increase in the percentage of vaginal deliveries with only one year of intervention can be considered a promising result for the project. The PPA is now in its fourth year of activity and has since continued to expand, incorporating another 100 private hospitals after the end of this period [14]. A continuous six year (2014–2019) increase in the percentage of vaginal deliveries was observed in one of the hospitals participating in the PPA, which also identified an important decrease in the average of NICU admissions, from 19.2% to 13.2% [22].

In the component “Reorganization of Care”, the increase in the percentage of vaginal deliveries were most significant for one intervention activity: the adoption of best practices in childbirth care. This was especially the case in pregnant women who underwent labor, showing its importance in promoting vaginal delivery and reducing intrapartum CS. The best practices evaluated included the provision of oral fluids, freedom of movement, access to a shower, and the use of non-pharmacological methods to manage pain. The effect of these practices in favoring vaginal delivery has already been documented in the literature [23] and was corroborated in this study, as they increased the probability of vaginal delivery by 50% when compared to women who did not use them. In addition to their physiological effects, these practices address the need for social support during childbirth, a feature recognized as being important for reproductive success [24]. In the context of deliveries occurring in a hospital setting, health professionals, mainly obstetric nurses, and doulas, become the source of social support.

In the overall assessment of each component of the PPA, the greatest difference in the probability of vaginal delivery between the woman participating and not participating in the project was observed in the “Women's participation” component. Women's participation was assessed through access to information, preference for the type of delivery at the end of pregnancy, participation in an antenatal group, visiting the maternity ward prior to their delivery, receiving information about the PPA, and preparing a birth plan. This is in line with recent publications that highlight activities aimed at women and their families as non-clinical interventions to reduce unnecessary CS and that focus on the importance of involving women in the formulation and implementation of childbirth care models based on their needs [23, 25]. This component had low implementation, which may be due to the difficulty in changing ingrained practices of women regarding care during their delivery/pregnancy [26]. A qualitative study evaluating changes in the work routine and methods of assisting women during labor in 8 hospitals participating in the PPA, highlighted the importance of offering the best information and communication channels to women. In their reports, health professionals pointed out the strengthening of women's autonomy through making shared decisions, resulting in the reduction of CS performed based on professional convenience [27].

The model of childbirth care in the Brazilian private sector, in which CS is a major component, is one that encourages the passivity of the woman, who remains lying down and anesthetized so that the birth of her child is the sole responsibility of the medical team. There is little encouragement for women's autonomy in seeking out information about the physiology of childbirth, the consequences of a CS, and the benefits of labor and vaginal delivery. However, even in the context of a quality improvement project, changes in these practices are not easy. For example, although the discussion about the type of delivery among groups of pregnant women during prenatal care has been shown to be an effective non-clinical intervention to reduce CS [25], only 40% of women in the group exposed to the PPA participated in prenatal care groups, as shown in Table 1.

One of the explanations for the increase in CS in Brazil is the preference of women for this type of delivery. In fact, a 2011 nationwide study conducted in Brazil showed a greater preference for delivery via CS in women in the private sector, especially in multiparous women with a previous CS [28], and a reduction in preference for a vaginal delivery throughout the pregnancy. Women who prefer a vaginal delivery at the end of pregnancy have a higher probability of vaginal delivery [28], and it is important to ensure access to information during pregnancy to allow an informed choice. Participation in the PPA increased the preference for vaginal delivery in late pregnancy [29]. However, the main factor associated with the preferred type of delivery at the end of pregnancy was the preference for the type of delivery at the beginning of pregnancy. The main reason cited for a preference for CS is the fear of a vaginal delivery.

The reduction in CS as a public policy should be supported by mass campaigns, explaining the risks of CS without clinical reasons for the woman and baby as well as the advantages of vaginal delivery. Furthermore, it is necessary to emphasize the risks of scheduling a delivery, which has contributed to the lower gestational age at birth in Brazil, when compared to other countries. This is mainly due to the excess of early-term births. It is known that pregnant women pay more attention to health campaigns, especially when the information relates to the health of their children. An example of this would be the mass campaigns in Brazil to promote breastfeeding. This campaign was very effective, as it had sensitive, aesthetic content showing the benefits of this practice for the future lives of women’s babies [30]. The lack of mass campaigns and public awareness was highlighted by a qualitative study involving 102 women who delivered in two hospitals participating in the PPA. Despite “Women's participation” being a central component of the project, the qualitative study showed that the communication channels established between women and hospitals are still fragile, limiting the possibility for women to drive change in attitudes towards vaginal delivery [4]. Strengthening the spaces for dialogue and enabling women to contribute with suggestions for the project may expand the number of allies and adherence to the activities of the project, as well as foster further improvements.

In the analysis of scenarios combining different activities to reduce CS rate, three scenarios showed a probability of 80% of vaginal delivery (scenarios 10, 11, and 12). All of them included not scheduling the delivery, having access to information about best practices during prenatal care, having access to at least 4 best practices during labor, and having their birth plan respected by the health professionals. The scenarios differed only by the team involved (hospital or external team) and by the presence of a nurse-midwife. It should be noted that an increase in the probability of vaginal delivery from 58 to 72% (S6) and 73% (S7) was observed in scenarios with fewer activities, but with the inclusion of access to best practices, reinforcing the importance of this activity in promoting vaginal delivery.

The participation of nurse-midwives in labor and birth care in the hospitals involved in the PPA was low, with 54.8% of women being assisted by a nurse-midwife during labor and only 2.2% during vaginal deliveries [31]. Surprisingly, removing nurse-midwives from the care team did not change the likelihood of having a vaginal delivery, provided other activities, such as access to best practices during labor, were in place. This may be explained either by the greater incorporation of best practices during labor by physicians, the low autonomy of nurses when attending to women in labor, making their performance less effective than expected, or both. A nationwide study conducted in Brazil evaluating the use of best practices during labor care in the private sector in 2017 found that, compared to 2011, there was an increase in the usage of best practices during labor and delivery [32]. On the other hand, the barriers and difficulties to the performance of nurse-midwives are undeniable, especially in the private sector, in which the physicians play a predominant role, as seen in this study for the standard of care group [33–35]

To discuss the findings of this study, it is also necessary to remember that complex interventions must be considered within the context in which they occur [36]. The PPA involved 29 activities, some related to lifestyle, and others related to psychological aspects and ideologies of users and health professionals, which may have influenced adherence to the intervention. Others are related to power interactions and intra-hospital hierarchy, making it difficult to measure their impact on vaginal delivery.

All these aspects may have affected the low degree of implementation of the planned activities during the first year of the PPA as shown by Torres et al. [26]. The implementation score of the component “Reorganization of Care” and “Women's Participation” was around 30%, which is far from ideal, but even with this low level of implementation, they still had an impact on reducing CS. This makes us wonder about the impact these components could have had if fully implemented.

A change in the culture, practices, and power dynamics surrounding childbirth is necessary to promote women's autonomy during labor and birth care. Women's birth plans may be seen by some as an affront to the technical knowledge of obstetricians. An environment with more horizontal interaction between women and health professionals is not widely adopted by many hospitals in Brazil, especially if the model of care is predominantly physician-centered. These changes do not happen quickly, as they demand time, work, discussion, and deep reflection from all stakeholders. Therefore, educational programs for health professionals would be welcome. Such programs should not only focus on the risks of CS and the benefits of vaginal birth for mothers and their babies but also make women aware of the high costs of the current model based on CS [37, 38]. These educational programs should also emphasize the importance of creating a system of care that brings joy to both healthcare professionals and mothers and their families.

According to Kingdon et al., changes in the behavior of health professionals and policymakers require three key facets: first, professionals being convinced that they are performing CS unnecessarily and vaginal delivery has an intrinsic value; second, discussion amongst intra- and interprofessional groups and agreements on how to change local norms and practices in various settings of labor and birth; and third, being able to deal with barriers, including the status and power of professional groups, quality of doctor-patient relationships, medico-legal issues, monetary gain and efficiency aspects [39].

In the process of changing attitudes towards a major public problem, the participation of peers and institutions with recognized prestige and authority among the members of the group that is expected to adhere to innovation is essential. This strategy was successfully used at the beginning of the PPA when socially respected members in Brazil participated in the invitation strategy used by the PPA coordination team. This invitation strategy was highly valued by the hospital's leaders and encouraged adherence to the PPA program [27]. The support of recognized institutions and leaders may encourage changes in the behavior of professionals, and it can be expected that this strategy will also be relevant throughout the entire process of implementation and sustainable use of recommended practices.

This study has some limitations. We restricted our analysis to Robson groups 1–4 to increase comparability of women assisted in the “PPA model of care” and in the “Standard of care model”. In addition, from phase 2 onwards, all hospitals adopted Robson´s group 1–4 as the target population, increasing interest in the effect of the PPA on this specific population. However, women in Robson´s group 5, who represent a third of C-sections in Brazilian private hospitals [3], were not evaluated, which is an important limitation. Strategies to reduce c-sections and improve maternal and perinatal outcomes for women with previous c-sections are of great interest to the scientific and clinical practitioners' community and should be included in future evaluative research. We were not able to evaluate the effects of “Governance” and “Monitoring” on the probability of promoting a vaginal delivery, as the changes related to these components were assessed at the level of the whole hospital rather than individual women. Future analyzes using the qualititative component will address the importance of these components. Finally, this evaluation does not include public hospitals. The private sector in Brazil is primarily responsible for the high rates of cesarean sections. The PPA model was developed with the particularities of this sector in mind, suggesting changes in key characteristics of CS practices in private hospitals. While the PPA could potentially be adapted for use in the public sector, modifications might be necessary. This study emphasizes that 'Reorganization of Care' is a fundamental component of the PPA, highlighting the need for tailored intervention strategies in public versus private hospital settings."

One of the strengths of our research was the building of a theoretical model—“The Birth Network”—with the participation of a wide variety of professionals including nurses, obstetricians, epidemiologists, and statisticians. The other strength is the statistical analysis through the Bayesian method which allowed us to compare the groups "Exposed to the PPA" and "Standard of Care” while isolating the effect of each activity and each component of the PPA on the probability of vaginal delivery. In addition, the BN method allowed simulations of different scenarios for the implementation of various activities to improve childbirth care.

This initiative from Brazil to improve the quality of birth care and reduce CS rates may be of interest to other middle-income countries in Latin America, Asia, Africa, and Oceania. This is because these countries have also shown an increase in CS rates between 2000 and 2020, especially within the private sector [10, 11]. Even though each of them has its context for this phenomenon, some aspects of the Brazilian case may be similar to other countries and may contribute to the complex task of reducing CS in any context.

## Conclusion

The PPA has been shown to be an effective quality improvement program, increasing the likelihood of vaginal delivery in private hospitals in Brazil. The “Reorganization of Care” component, in particular the use of best practices during labor and birth care, contributed the most to increasing the likelihood of vaginal delivery. The combination of not scheduling a CS before labor; allowing pregnant women to access information about the best practices during prenatal care; implementing at least 4 best practices during labor; and respecting the birth plan of women, together resulted in a higher probability of vaginal delivery. The results of the network show that there are different possibilities for combining activities to reduce CS, which may inform policymakers and be used to prioritize future interventions.

## Data Availability

The datasets used and/or analyzed in the current study are available from the corresponding author upon reasonable request.

## Abbreviations

ANS: National Agency for Supplementary Health
BN: Bayesian Network
CS: Caesarean section
HIAE: Hospital Israelita Albert Einstein
IHI: Institute for Healthcare Improvement
PPA: “Projeto Parto Adequado” (Adequate Childbirth Project)
